# Effects of internet-based cognitive behavioral therapy on postpartum depression: A protocol for systematic review and meta-analysis

**DOI:** 10.1097/MD.0000000000028964

**Published:** 2022-03-04

**Authors:** Fang Wang, Hongcheng Zhu, Xiaoju Yang, Fang Liao

**Affiliations:** 1 Department of Obstetrics and Gynaecology, The National Hospital Of Enshi Autonomous Prefecture, Enshi, Hubei Province, China.

**Keywords:** cognitive behavioral therapy, meta-analysis, online program, postpartum depression, protocol

## Abstract

**Background:**

Postpartum depression is one of the most common complications during the postpartum period. In recent years, internet-based psychological interventions have made significant progress and provided a new psychotherapy model. Internet-based cognitive behavioral therapy (ICBT) for postpartum depression has achieved good results. However, the effectiveness of ICBT for postpartum depression reported by different studies still remains inconsistent. Therefore, a meta-analysis was used to further evaluate the efficacy of ICBT for postpartum depression, aiming to provide evidence to support nonpharmacological intervention strategies in the clinic.

**Methods:**

The databases of PubMed, Web of Science, Scopus, Cochrane Library, Embase, China Scientific Journal Database, China National Knowledge Infrastructure, Chinese Biomedical Literature Database, and Wanfang Data will be searched. The randomized controlled trials of ICBT will be included for postpartum depression published before February 2022. Two independent researchers will independently complete literature selection, risk of bias assessment and data extraction. The disagreements will be discussed with a third party for the final decision. Cochrane Risk of Bias Assessment Tool will be used for literature quality assessment. Data processing will be conducted by RevMan 5.4 software.

**Results:**

The results of this meta-analysis will be submitted to a peer-reviewed journal for publication.

**Conclusions:**

For the question whether ICBT for postpartum depression is efficacy, this study can provide more comprehensive and strong evidence.

**Ethics and dissemination:**

The ethical approval was not required for this study. The systematic review will be published in a peer-reviewed journal, presented at conferences, and shared on social media platforms.

**OSF REGISTRATION NUMBER:**

DOI 10.17605/OSF.IO/EQJDH.

## 1 Introduction

Postpartum depression is defined as women's significant depressive symptoms or typical depressive episodes during the puerperium, characterized by low energy, extreme sadness, irritability, and suicidality.^[1–3]^ It has been reported that 10% to 20% of foreign women have various degrees of depressive symptoms during the perinatal period.^[4,5]^ The average prevalence of postpartum depression in China is 14.7%.^[6]^ Postpartum depression can lead to cognitive, behavioral, and emotional impairment in mothers and has long-term negative effects on children.

Now, medication is widely chosen to prevent maternal depression: More than 7% of mothers used antidepressants and at least 75% of those diagnosed with depression were recommended antidepressants.^[7,8]^ However, mothers choose to refuse medication concerning the safety of the medication for their infants. Among those who do receive antidepressants, there is a discontinuation rate of over 50% and a relapse rate of 70%.^[9]^ Therefore, it is particularly important to find a safe and effective intervention strategy.

As a group of short course psychotherapy methods, cognitive behavioral therapy (CBT) can adjust individual's poor cognition and eliminate bad mood by changing thinking and behavior, with cognitive reconstruction and behavior modification as the core.^[10,11]^ CBT is effective in treating and preventing postpartum depression.^[12]^ Currently, there are several barriers to the implementation of CBT, including lack of professional therapists, remote location, long waiting time, and high cost.^[13]^

In recent years, internet-based psychological interventions have made significant progress and offered a new model of psychotherapy. Internet-based psychological interventions have the characteristics of high flexibility and accessibility, privacy protection, and lower cost.^[14]^ Internet-based cognitive behavioral therapy (ICBT) is considered to be an effective treatment for depression. However, with the increasing research on ICBT in postnatal depression, the effectiveness of ICBT for postpartum depression reported by different studies still remains inconsistent.^[12,15–18]^ Therefore, a meta-analysis will be used to further evaluate the efficacy of ICBT for postpartum depression and provide evidence to support nonpharmacological intervention strategies in the clinic.

## 2 Methods

### 2.1 Study registration

The protocol of this review was registered in Open Science Framework (OSF) (OSF registration number: DOI 10.17605/OSF.IO/EQJDH). Besides, it was reported as per the statement guidelines of preferred reporting items for systematic reviews and meta-analysis protocol.^[19]^

### 2.2 Inclusion criteria for study selection

#### 2.2.1 Types of studies

Randomized controlled trials (RCTs) of ICBT for postpartum depression will be included.

#### 2.2.2 Types of participants

Postpartum women who were at the age of ≥18 years and met the diagnostic criteria of depressive mental disorder or depression score scale.

#### 2.2.3 Types of interventions

Patients in the control group used the standard care protocol. In the experiment group, ICBT was performed on this basis, including WeChat, APP, Messages, telephone, e-mail, etc.

#### 2.2.4 Types of outcome indexes

Depression-related scales include Hamilton depression rating scale, Beck depression inventory, self-rating depression scale, and Edinburgh postpartum depression scale.

### 2.3 Exclusion criteria

1) The literature data are incomplete;
2) Republished literature;
3) Editorials, letters, reviews, pharmacological or chemical experiments, etc.

### 2.4 Data sources

PubMed, Web of Science, Scopus, Cochrane Library, Embase, China Scientific Journal Database, China National Knowledge Infrastructure, Chinese Biomedical Literature Database, and Wanfang Data for RCTs on ICBT for postpartum depression were searched on computer and conducted from the time of database creation to February 2022. In addition, unpublished related studies in the clinical trial registry were searched and references to the included literature were traced to ensure the recall rates. The retrieval strategy is formulated by combining MeSH terms with free words. Literature retrieval strategies are shown in Table 1, taking PubMed database as an example.

**Table 1:** Search strategy in PubMed database.

Number	Search terms
#1	Depression, postpartum[MeSH]
#2	Postnatal depression[Title/Abstract]
#3	Postpartum depression[Title/Abstract]
#4	Post-natal depression[Title/Abstract]
#5	Post-partum depression[Title/Abstract]
#6	Depression, post-natal[Title/Abstract]
#7	Depression, post-partum[Title/Abstract]
#8	Depression, postnatal[Title/Abstract]
#9	Post natal depression[Title/Abstract]
#10	Post partum depression[Title/Abstract]
#11	or/1–10
#12	Cognitive therapy[MeSH]
#13	Behavior therapy, cognitive[Title/Abstract]
#14	Psychotherapy, cognitive[Title/Abstract]
#15	Cognition therapy[Title/Abstract]
#16	Cognitive behavior therapy[Title/Abstract]
#17	Cognitive behavioral therapy[Title/Abstract]
#18	Cognitive psychotherapy[Title/Abstract]
#19	Therapy, cognition[Title/Abstract]
#20	Therapy, cognitive[Title/Abstract]
#21	Therapy, cognitive behavior[Title/Abstract]
#22	Behavior therapies, cognitive[Title/Abstract]
#23	Behavioral therapies, cognitive[Title/Abstract]
#24	Behavioral therapy, cognitive[Title/Abstract]
#25	Cognition therapies[Title/Abstract]
#26	Cognitive behavior therapies[Title/Abstract]
#27	Cognitive behavioral therapies[Title/Abstract]
#28	Cognitive psychotherapies[Title/Abstract]
#29	Cognitive therapies[Title/Abstract]
#30	Psychotherapies, cognitive[Title/Abstract]
#31	Therapies, cognition[Title/Abstract]
#32	Therapies, cognitive[Title/Abstract]
#33	Therapies, cognitive behavior[Title/Abstract]
#34	Therapies, Cognitive Behavioral[Title/Abstract]
#35	Therapy, cognitive behavioral[Title/Abstract]
#36	or/12–35
#37	Online[Title/Abstract]
#38	Internet[Title/Abstract]
#39	Web[Title/Abstract]
#40	Computer[Title/Abstract]
#41	Mobile[Title/Abstract]
#42	APP[Title/Abstract]
#43	WeChat [Title/Abstract]
#44	Letter[Title/Abstract]
#45	Mail[Title/Abstract]
#46	E-mail[Title/Abstract]
#47	Email[Title/Abstract]
#48	Messages[Title/Abstract]
#49	Phone [Title/Abstract]
#50	or/37–49
#51	Randomized controlled trial[MeSH]
#52	Random∗[Title/Abstract]
#53	Clinic trial [Title/Abstract]
#54	or/51–53
#55	#11 and #36 and #50 and #54

### 2.5 Data collection and analysis

#### 2.5.1 Data extraction and management

**Figure 1:**
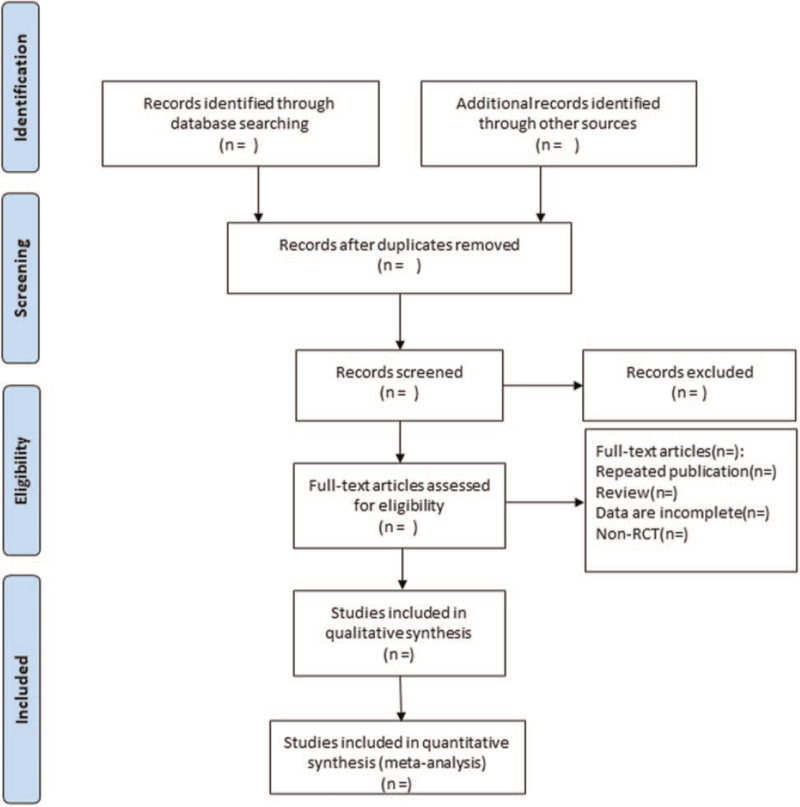
Flow diagram of study selection process.

According to the inclusion and exclusion criteria, 2 researchers independently screened the literature, extracted data and checked them. In case of disagreement, a third party will discuss and resolve the issue. The literature screening consisted of 3 basic steps: initial screening: reading the title and abstract to eliminate the obvious ineligible literature; full-text screening: further reading the full text to decide whether to include the literature; and obtaining more information before deciding the selection of the literature with doubts or disagreements. The extracts include basic information about the included studies, characteristics of the study population, details of risk of bias evaluation, interventions, outcome indicators, etc. The screening flow chart of this study is presented in Figure 1.

#### 2.5.2 Assessment of risk of bias

The quality of RCT was evaluated using the RCT bias risk assessment tool recommended in Cochrane Handbook 5.1.0.^[20]^ The evaluation of the main points includes random sequence generation, allocation hiding, blinding, integrity of outcome data, selective reporting of study results, and other biases. The evaluation results will be classified into the high-risk, low-risk, and unclear categories.

#### 2.5.3 Measures of treatment effects

Continuous variables will be combined using the standardized mean differences and corresponding 95% confidence intervals.

#### 2.5.4 Management of missing data

In case of any missing data in relevant study, the original data will be requested by email. If there is a failure in the data request, such data shall be excluded from this study.

#### 2.5.5 Assessment of heterogeneity and data synthesis

Statistical analysis of the included RCTs will be performed with the RevMan 5.4 software. The heterogeneity will be assessed by the I^2^ test. I^2^ ≤ 50% is considered as a small heterogeneity, and a fixed-effect model will be used to combine the effect size, otherwise, a random-effects model will be introduced.

#### 2.5.6 Assessment of reporting biases

If there are more than 10 trials in the meta-analysis, qualitative checks will be conducted via funnel plots to analyze potential reporting bias.^[21]^

#### 2.5.7 Subgroup analysis

Subgroup analysis will be performed based on disease severity, type of ICBT, and duration of intervention.

#### 2.5.8 Sensitivity analysis

To test the stability and reliability of the results of the meta-analysis, subgroup analysis will be performed using a one-by-one elimination method.

#### 2.5.9 Ethics and dissemination

The contents of this paper do not involve moral approval or ethical review and will be presented in print or at relevant conferences.

## 3 Discussion

CBT, one of the most widely used psychotherapies with evidence-based applicability, is a structured, short-course, effective, cognitively oriented psychotherapy.^[22]^ Currently, the main treatments for postpartum depression include pharmacotherapy, psychotherapy, physical therapy, and other complementary treatments. Among them, CBT has a wide range of application, without toxic side effects. It can improve patients’ dysfunctional cognition, with good long-term effects.^[23]^

Related researchers’ early attempts to use internet-provided interventions to reduce depressive symptoms have yielded ambiguous results.^[24,25]^ However, subsequent studies reported more promising findings.^[26–29]^ Notably, some encouraging results have been obtained using ICBT interventions for other mental health disorders, including the treatment of panic disorder, post-traumatic stress disorder, and complicated grief.^[30–34]^ ICBT offers significant user convenience by allowing more maternal choice, reducing transportation and wait times, and protecting patient privacy.^[18]^

Currently, there are growing researches on the use of ICBT for postpartum depression. However, there are inconsistencies in the efficacy of ICBT for postpartum depression reported in different literatures. This study used a meta-analysis to further evaluate the efficacy of ICBT for postpartum depression, aiming to provide a high level of evidence-based medicine for the management of postpartum depression.

## Author contributions

**Conceptualization:** Fang Liao, Fang Wang.

**Data curation:** Fang Wang, Hongcheng Zhu.

**Formal analysis:** Hongcheng Zhu.

**Funding acquisition:** Fang Liao.

**Investigation:** Hongcheng Zhu.

**Methodology:** Hongcheng Zhu.

**Project administration:** Fang Liao.

**Resources:** Hongcheng Zhu, Xiaoju Yang.

**Software:** Hongcheng Zhu, Xiaoju Yang.

**Supervision:** Fang Liao.

**Validation:** Xiaoju Yang.

**Visualization:** Xiaoju Yang.

**Writing – original draft:** Fang Liao, Fang Wang.

**Writing – review & editing:** Fang Liao, Fang Wang.
